# Supplementary material for: The Genetic Diversity of Mink (Neovison vison) Populations in China
Source: Animals (Basel). 2023 Apr 27;13(9):1497. doi: 10.3390/ani13091497 (PMC10177056; doi:10.3390/ani13091497)
Supplement: Supplementary file 1 [file animals-13-01497-s001.zip › animals-2247948-supplementary.pdf]

animals

MDPI

Article

The Genetic Diversity of Mink (*Neovison vison*) Populations in China

Tietao Zhang, Hu Li, Peter Forge Larsen, Hengxing Ba, Hongyu Shi, Haihua Zhang and Zongyue Liu

Table S1. List of quality filtering of reads on 97 American mink genomes.

Sample	HQ_Reads	HQ_Reads (%)	HQ_Data(bp)	HQ_Data (%)
BLM1	27590406	95.62	3866732627	94.37
BLM3	27963750	95.5	3917848859	94.23
BLM4	28079484	63.99	3933245118	63.17
BLM6	37283864	94.97	5210163878	93.52
BLM8	27639292	95.45	3870846532	94.15
BLM10	29227434	78.7	4092900125	77.86
BLM11	29162680	95.19	4083851342	93.88
BLM12	35011814	95.45	4885007444	94.13
BLM13	27637878	95.5	3871742031	94.22
BLM14	31626634	95.83	4433900435	94.56
BLM15	28658734	94.83	4008655686	93.41
BLM21	35305254	95.49	4945124564	94.18
BLM22	30344232	95.89	4254427172	94.71
BLM23	29767944	96.61	4179745583	95.55
BLM24	16450848	94.55	2294882393	92.86
A01	15452570	95.06	2157501060	93.45
A31	29684046	95.56	4158119489	94.29
A28	27543006	95.75	3860170181	94.52
A29	28205558	95.82	3952757584	94.59
A505	30945558	95.38	4333608947	94.09
A506	29625876	95.38	4149659124	94.1
A504	30618962	95.93	4292737260	94.73
A539	20304554	95.4	2845279851	94.17
A509	22007740	95.4	3083798181	94.16
A511	22636244	95.29	3171022699	94.03
A517	21880776	94.89	3062373030	93.55
A519	22258602	95.43	3118709545	94.18
B209	20474692	95.05	2867416326	93.77
B210	21969328	94.97	3076181441	93.67
B211	20248638	95.09	2835150298	93.78
Y1	24813900	95.69	3479233033	94.51
Y2	24905734	95.99	3494462757	94.87
Y3	23224870	96	3258540819	94.88
Y7	25443648	95.8	3568174101	94.63
Y8	26179448	95.96	3672419548	94.82
Y9	24836028	95.84	3483845303	94.7
Y10	28179248	95.82	3952711216	94.67
RM43	25271742	93.95	3533302975	92.41
RM45	24951458	94.74	3489924760	93.37
RM52	25630462	94.69	3584218161	93.31

RM4	24318668	95.22	3404470828	93.93
RM9	23140928	95.01	3238381262	93.68
RM10	24372992	95.02	3410480143	93.69
K1	23543102	94.1	3288035049	92.61
K6	25303048	94.37	3536306806	92.94
K7	24708948	94.47	3452788353	93.02
K8	24354604	94.81	3406811760	93.45
K10	25920758	95.02	3627582936	93.7
A5404	28361638	94.81	3967045289	93.45
A5372	25212842	95.12	3529118817	93.82
A5361	25009122	94.6	3495729259	93.18
A5263	25397482	94.88	3552473079	93.52
A5240	23223132	94.44	3245694085	93
A5392	23424444	95.66	3282282045	94.45
A5237	21090448	95.71	2955336988	94.51
A5232	20285326	95.79	2842769017	94.59
A5149	22306176	95.86	3126840811	94.68
A5222	21198950	96.01	2972346569	94.86
A5257	22117806	95.88	3100829161	94.71
A5266	23002176	96.19	3226855459	95.09
A5279	27350118	96.12	3835225540	94.97
B2205	22636152	95.72	3171182749	94.48
B2206	27044368	93.85	3778886538	92.37
B2141	26858530	93.95	3753963785	92.5
B2224	26970998	93.85	3767371938	92.34
B2258	28427676	92.92	3963455662	91.25
B2259	27548820	93.32	3845341922	91.75
B2282	27817006	95.11	3895511099	93.82
B2122	30071908	92.99	4194818642	91.36
B2120	25854844	93.6	3610257712	92.06
B2119	30619896	95.17	4288973436	93.9
B2108	27506062	94.4	3847149122	93
B2104	28578490	94.89	4000935539	93.57
B2100	29331928	95.75	4113949435	94.59
B2304	28701638	95.37	4022319464	94.14
K30	29879272	94.58	4182553297	93.25
K60	31076126	93.94	4344332976	92.48
K70	30401322	94.68	4256970520	93.37
K80	28968120	95.52	4062218928	94.34
K90	27810138	95.18	3897824005	93.95
K100	32590676	94.62	4561224353	93.27
Y40	28909758	95.31	4051787651	94.08
Y70	28419412	95.91	3987707730	94.79
Y80	28017408	95.2	3927768635	94
Y90	28326508	95.85	3974572117	94.74
Y100	26222306	95.7	3678741396	94.56
H1	35170182	94.37	4918846973	92.98
H2	30831362	94.26	4311657684	92.87
H3	29868156	94.41	4177401525	93.03
H5	29095292	94.42	4069469186	93.04
H7	27872206	94.53	3899725297	93.18

H8	28278582	93.97	3951918863	92.52
H9	31392950	94	4387211644	92.55
H10	28355530	94.07	3963750384	92.65
H11	26383146	93	3679922517	91.38
H12	25849770	92.99	3605822062	91.39
B272	26190554	93.15	3653580604	91.54
total	2590562736	9168.9	362626920124	9040.99
average	26706832.3	94.52	3738421857	93.21

Note: HQ Reads：High-quality reads number; HQ Data (bp): Number of high-quality reads-bases; HQ Reads (%): the percentage of high-quality reads to the lower machine reads; HQ Data (%): the percentage of high-quality sequence bases in the lower machine base.

Table S2. List of sequencing data alignment on 97 American mink genomes.

Sample	Total_reads	Mapped_reads	Mapping_rate
BLM12	35336875	34944831	98.89
Y2	25129808	24884571	99.02
BLM15	28907080	28581309	98.87
B211	20448011	20217953	98.87
A5361	25265557	24986908	98.90
RM4	24561842	24291532	98.90
B2108	27749846	27445184	98.90
A29	28485078	28162404	98.87
Y10	28463217	28111895	98.77
B2258	28674855	28361800	98.91
K100	32873244	32507394	98.89
A511	22856562	22586693	98.82
B2205	22868735	22602074	98.83
A5266	23219924	22987797	99.00
BLM1	27832357	27523364	98.89
H3	30115843	29774709	98.87
A5232	20484591	20263633	98.92
H11	26611644	26307713	98.86
A5222	21395934	21163447	98.91
BLM6	37678530	37238528	98.83
BLM8	27883821	27534043	98.75
BLM22	30630061	30267088	98.81
B272	26407392	26108788	98.87
A506	29906386	29586688	98.93
K90	28054764	27764058	98.96
H9	31674155	31302277	98.83
B2122	30354753	29992781	98.81
B2206	27286265	27004619	98.97
K80	29227174	28874438	98.79
H7	28116024	27787353	98.83
A5404	28627162	28309599	98.89
BLM21	35637368	35228765	98.85
H12	26061893	25763455	98.85
A505	31243679	30891463	98.87
Y70	28665863	28347325	98.89
Y8	26418811	26134366	98.92
K7	24965526	24664905	98.80

A5372	25451466	25197294	99.00
Y1	25053309	24801786	99.00
RM45	25201782	24908925	98.84
Y100	26469724	26137970	98.75
Y40	29152322	28317839	97.14
RM9	23370061	23084975	98.78
RM52	25880556	25579951	98.84
B2141	27109192	26830083	98.97
BLM11	29425631	29071386	98.80
B2304	28986079	28654337	98.86
BLM13	27894669	27563988	98.81
A5392	23644017	23399801	98.97
B2224	27208476	26888278	98.82
B2119	30894208	30558347	98.91
B2100	29615825	29292721	98.91
B2282	28080585	27774269	98.91
A31	29973942	29632966	98.86
B210	22175455	21927918	98.88
BLM14	31960601	31565441	98.76
A28	27814732	27493796	98.85
B209	20675908	20451608	98.92
A5257	22320279	22068241	98.87
A5240	23451287	23217687	99.00
Y3	23453866	23223331	99.02
A539	20503183	20278578	98.90
H2	31094699	30746301	98.88
BLM23	30062916	29706069	98.81
H5	29344893	29011023	98.86
K60	31350809	30990645	98.85
K70	30676227	30175133	98.37
B2120	26091672	25833117	99.01
Y80	28242857	27921913	98.86
B2259	27785558	27494880	98.95
H10	28595936	28277020	98.88
Y90	28573569	28272409	98.95
BLM24	16585945	16413491	98.96
A5279	27585206	27226811	98.70
A517	22095355	21865369	98.96
A519	22478774	22230946	98.90
A509	22220172	21981493	98.93
A01	15573858	15404246	98.91
B2104	28833372	28553225	99.03
K6	25551825	25272200	98.91
BLM10	29472041	29135876	98.86
RM43	25515200	25217951	98.84
Y9	25075264	24794248	98.88
K8	24585870	24320883	98.92
RM10	24610027	24311873	98.79
K10	26168459	25876138	98.88
Y7	25688454	25417648	98.95
K1	23773464	23498601	98.84

H1	35465506	35069621	98.88
K30	30147496	29800829	98.85
BLM3	28221006	27864912	98.74
H8	28515363	28192581	98.87
A504	30908395	30578982	98.93
BLM4	28336316	27999093	98.81
A5263	25646482	25359329	98.88
A5237	21291742	21099056	99.10
A5149	22518415	22294881	99.01
The average of Mapping_rate			98.86

Note: Total Reads: total number of sequences; Mapped reads: number of sequences that can be aligned to the reference genome; Mapping Rate (%): the proportion of the number of sequences aligned to the reference genome to the total number of sequences

Table S3. List of sequencing data quality on 97 American mink genomes.

Sample	Reads_Num	Total_Bases(bp)	GC(%)	Q20(%)	Q30(%)
BLM1	28855216	4097235140	51.83	97.37	93.28
BLM3	29280492	4157644666	51.77	97.3	93.14
BLM4	43878624	6226785807	50.07	97.15	92.69
BLM6	39257662	5571005674	50.38	97.05	92.56
BLM8	28956410	4111512661	52.26	97.27	93.08
BLM10	37136316	5256986097	50.91	97.21	92.87
BLM11	30636492	4350156081	51.98	97.18	92.89
BLM12	36679392	5189725117	49.89	97.24	92.89
BLM13	28941290	4109396396	51.13	97.32	93.16
BLM14	33003692	4688777519	51.88	97.44	93.44
BLM15	30220778	4291324836	51.47	97.04	92.64
BLM21	36972422	5250832024	50.52	97.28	93.04
BLM22	31643330	4492248813	51.33	97.61	93.93
BLM23	30813638	4374493601	51.82	97.9	94.5
BLM24	17399208	2471367137	50.79	96.76	91.83
A01	16254954	2308749042	51.18	96.95	92.24
A31	31064334	4410134399	51.18	97.48	93.68
A28	28766298	4083870717	51.18	97.56	93.83
A29	29435036	4178781732	51.13	97.56	93.82
A505	32443674	4605931532	51.18	97.41	93.54
A506	31062252	4409976243	51.25	97.41	93.53
A504	31918984	4531472642	51.44	97.63	93.96
A539	21282866	3021455183	51.87	97.49	93.74
A509	23069676	3275093250	51.81	97.48	93.7
A511	23755242	3372427321	51.56	97.43	93.62
A517	23058278	3273462269	51.23	97.27	93.31
A519	23325012	3311330288	51.73	97.47	93.68
B209	21540192	3057980354	51.61	97.35	93.47
B210	23131910	3284023720	51.63	97.31	93.38
B211	21294568	3023125749	51.43	97.35	93.46
Y1	25932132	3681468998	51.68	97.58	93.88
Y2	25946120	3683462941	52.1	97.7	94.12
Y3	24192164	3434455148	52.21	97.71	94.14
Y7	26559198	3770508878	51.86	97.62	93.98
Y8	27281850	3873090528	52.83	97.68	94.08

Y9	25913406	3678813920	52.22	97.65	94.02
Y10	29409328	4175276842	52.17	97.64	93.99
RM43	26898702	3823378977	52.21	96.89	92.55
RM45	26337062	3737736140	51.61	97.19	93.07
RM52	27066360	3841248111	51.75	97.15	92.98
RM4	25539242	3624477802	52.94	97.38	93.48
RM9	24357136	3456716522	51.7	97.28	93.25
RM10	25650428	3640227718	53.25	97.29	93.31
K1	25018070	3550488197	51.53	96.93	92.57
K6	26811192	3805054705	51.55	97.03	92.76
K7	26156720	3712044624	51.73	97.06	92.84
K8	25689020	3645610565	51.35	97.22	93.16
K10	27280696	3871456999	52.07	97.29	93.27
A5404	29913796	4245102619	52.17	97.22	93.18
A5372	26505404	3761427947	51.96	97.35	93.42
A5361	26436208	3751586187	51.73	97.11	92.96
A5263	26768642	3798768950	52.25	97.24	93.24
A5240	24591600	3489957564	51.48	97.07	92.9
A5392	24487284	3475047659	52.51	97.56	93.87
A5237	22035332	3127106929	52.12	97.6	93.98
A5232	21177968	3005405833	52.44	97.62	94
A5149	23270430	3302402516	51.75	97.65	94.04
A5222	22079778	3133438993	52.42	97.71	94.19
A5257	23069146	3273890199	52.81	97.67	94.12
A5266	23912468	3393603378	52.01	97.8	94.35
A5279	28455598	4038483349	54.95	97.76	94.38
B2205	23649118	3356347999	52.74	97.59	93.99
B2206	28816298	4091021618	51.6	96.87	92.48
B2141	28587138	4058519094	51.49	96.91	92.58
B2224	28739000	4080032763	52.34	96.86	92.45
B2258	30593184	4343297433	52.91	96.54	91.89
B2259	29521714	4191139597	51.71	96.67	92.09
B2282	29246784	4152102152	52.88	97.34	93.42
B2122	32339300	4591332762	51.36	96.53	91.84
B2120	27621686	3921533806	51.46	96.78	92.32
B2119	32174332	4567807764	52.53	97.38	93.4
B2108	29136572	4136528324	52.19	97.07	92.77
B2104	30117018	4275907783	51.69	97.25	93.1
B2100	30634128	4349182310	51.37	97.6	93.82
B2304	30095866	4272925541	51.69	97.46	93.57
K30	31590976	4485493907	51.21	97.17	92.98
K60	33082148	4697407870	51.15	96.91	92.47
K70	32108514	4559102032	51.41	97.22	93.09
K80	30327720	4305873537	52.32	97.54	93.74
K90	29219880	4148824108	51.98	97.4	93.44
K100	34442404	4890333372	51.49	97.17	92.97
Y40	30333014	4306941029	53.75	97.44	93.59
Y70	29632550	4206716388	51.73	97.65	93.89
Y80	29428702	4178327573	51.79	97.42	93.47
Y90	29551662	4195196725	51.63	97.66	93.92
Y100	27401136	3890357158	52.12	97.62	93.89

H1	37268910	5290028083	51.95	97.05	92.64
H2	32709550	4642766024	51.51	97.01	92.57
H3	31635972	4490335224	51.59	97.04	92.59
H5	30813290	4373711906	51.64	97.07	92.71
H7	29484462	4184994827	51.95	97.11	92.79
H8	30091952	4271214509	51.38	96.88	92.3
H9	33395528	4740481024	51.95	96.9	92.4
H10	30141820	4278385163	51.41	96.93	92.4
H11	28370488	4026929699	51.29	96.54	91.8
H12	27797616	3945633143	51.52	96.54	91.81
B272	28117476	3991042099	52.77	96.59	91.93
total	2748038626	390076820094	5024	9435.63	9042.09
average	28330295	4021410516	51.79	97.27	93.21

Note: Q20: the percentage of bases with a Phred value greater than 20 to the total bases; Q30: the percentage of bases with a Phred value greater than 30 to the total bases; GC(%): the percentage of G and C of the four bases in clean reads

Table S4. Indel test result statistics.

Sample	HOM_REF	HET	UNKNOWN	HOM_ALT
A01	59409	16791	107969	21564
A28	62048	17511	104362	21812
A29	61872	16866	104703	22292
A31	62473	17653	103315	22292
A504	64240	17315	100827	23351
A505	63010	18103	101548	23072
A506	63510	17114	102224	22885
A509	65709	17449	99036	23539
A511	65802	17681	98250	24000
A5149	65202	17153	99955	23423
A517	61895	16544	104872	22422
A519	65278	16552	100289	23614
A5222	68834	19170	92405	25324
A5232	63156	16087	104556	21934
A5237	63541	17081	102727	22384
A5240	62187	17713	103737	22096
A5257	70416	18684	90669	25964
A5263	63828	16982	102290	22633
A5266	59187	22244	103646	20656
A5279	62770	33169	87135	22659
A5361	62613	17867	102394	22859
A5372	69148	18400	93203	24982
A539	66797	17519	97175	24242
A5392	68277	18318	94669	24469
A5404	70387	21187	89123	25036
B209	64372	16836	101785	22740
B210	66999	18482	96477	23775
B2100	66388	18193	97087	24065
B2104	74755	20975	82756	27247
B2108	74458	23409	79792	28074
B211	63440	16692	103164	22437
B2119	75191	22639	78933	28970

B2120	70136	20494	89710	25393
B2122	67130	19253	94627	24723
B2141	65446	19150	98104	23033
B2205	64021	17045	101826	22841
B2206	71677	21093	86426	26537
B2224	73505	23223	81477	27528
B2258	75304	25775	75843	28811
B2259	71409	21703	86050	26571
B2282	69655	20085	90789	25204
B2304	65538	18263	98085	23847
B272	74114	24628	78868	28123
BLM1	69541	20411	90223	25558
BLM10	71946	23250	83644	26893
BLM11	71476	20454	87355	26448
BLM12	69170	26240	77364	32959
BLM13	65680	17746	98410	23897
BLM14	70981	26891	75213	32648
BLM15	69431	21621	89494	25187
BLM21	73518	27544	70063	34608
BLM22	65728	18607	98255	23143
BLM23	66544	17302	98643	23244
BLM24	59320	16517	108905	20991
BLM3	69739	20481	90075	25438
BLM4	69383	20502	90481	25367
BLM6	70788	28768	70612	35565
BLM8	71554	21872	86181	26126
H1	73034	24157	80219	28323
H10	69464	21513	88489	26267
H11	66014	20047	94629	25043
H12	69931	21876	87094	26832
H2	70285	22278	86391	26779
H3	70988	22329	85204	27212
H5	72094	22742	83201	27696
H7	72172	22138	83497	27926
H8	70506	22209	85791	27227
H9	71828	23064	83268	27573
K1	66561	17920	97470	23782
K10	70755	20493	89251	25234
K100	71989	23175	84254	26315
K30	68736	20623	90982	25392
K6	65822	18962	97596	23353
K60	68752	21003	91275	24703
K7	65960	18891	97650	23232
K70	71487	22357	86205	25684
K8	65467	18736	98221	23309
K80	71288	20343	88226	25876
K90	71312	20566	87934	25921
RM10	72194	21875	84839	26825
RM4	70601	21230	87871	26031
RM43	70115	21505	87766	26347
RM45	67719	19763	93984	24267

RM52	68312	20078	92442	24901
RM9	67941	19226	93930	24636
Y1	63439	15829	104888	21577
Y10	66573	17748	98836	22576
Y100	68562	17639	95688	23844
Y2	66880	16438	99362	23053
Y3	64464	15982	103281	22006
Y40	75533	25149	77117	27934
Y7	65918	16700	100202	22913
Y70	64369	16442	102335	22587
Y8	69214	18507	93835	24177
Y80	69781	18907	92632	24413
Y9	67396	17143	98207	22987
Y90	63833	15837	104070	21993

Note: HOM_REF: the sample is homozygous reference; HET: the sample is heterozygous; UNKNOWN; the sample is genotype missing; HOM_ALT: the sample is homozygous alternate.

Table S5. GO classification of the selected genes in BLM_mink and SM mink.

BLM			SM		
**GO.ID**	**Term**	**Pvalue**	**GO.ID**	Term	Pvalue
GO:0036452	ESCRT complex	0.02628439	GO:0042175	nuclear outer membrane-endoplasmic reticulum membrane network	0.00012882
GO:0031225	anchored component of membrane	0.029164258	GO:0044432	endoplasmic reticulum part	0.000147938
GO:0010008	endosome membrane	0.037755675	GO:0031984	organelle subcompartment	0.000203809
GO:0005615	extracellular space	0.042965728	GO:0012505	endomembrane system	0.0002707
GO:0044440	endosomal part	0.046275251	GO:0005783	endoplasmic reticulum	0.001466313
GO:0030176	integral component of endoplasmic reticulum membrane	0.065878048	GO:0008021	synaptic vesicle	0.003356567
GO:0031227	intrinsic component of endoplasmic reticulum membrane	0.065878048	GO:0070382	exocytic vesicle	0.003948276
GO:0005768	endosome	0.068647184	GO:0098793	presynapse	0.009307329
GO:0005085	guanyl-nucleotide exchange factor activity	0.001366893	GO:0016174	NAD(P)H oxidase activity	4.29E-05
GO:0019899	enzyme binding	0.002416696	GO:0050664	oxidoreductase activity, acting on NAD(P)H, oxygen as acceptor	0.000423097
GO:0004340	glucokinase activity	0.003623188	GO:0001540	amyloid-beta binding	0.000631932
GO:0004801	sedoheptulose-7-phosphate:D-glyceraldehyde-3-phosphate	0.003623188	GO:0004697	protein kinase C activity	0.001169546

	glyceronetransferase activity				
GO:0016744	transferase activity, transferring aldehyde or ketonic groups	0.003623188	GO:0042277	peptide binding	0.004256545
GO:0035299	inositol pentakisphosphate 2-kinase activity	0.003623188	GO:0005415	nucleoside:sodium symporter activity	0.006587615
GO:0051020	GTPase binding	0.005792961	GO:0010309	acireductone dioxygenase [iron(II)-requiring] activity	0.006587615
GO:0004967	glucagon receptor activity	0.007233547	GO:0004601	peroxidase activity	0.009912783
GO:0005152	interleukin-1 receptor antagonist activity	0.007233547	GO:0004379	glycylpeptide N-tetradecanoyltransferase activity	0.013132373
GO:0008158	hedgehog receptor activity	0.007233547	GO:0008124	4-alpha-hydroxytetrahydrobiopterin dehydratase activity	0.013132373
GO:0000291	nuclear-transcribed mRNA catabolic process, exonucleolytic	0.002904263	GO:0017000	antibiotic biosynthetic process	6.80014E-05
GO:0001960	negative regulation of cytokine-mediated signaling pathway	0.002904263	GO:0050665	hydrogen peroxide biosynthetic process	6.80014E-05
GO:0034427	nuclear-transcribed mRNA catabolic process, exonucleolytic, 3'-5'	0.002904263	GO:0042743	hydrogen peroxide metabolic process	0.000202904
GO:0060761	negative regulation of response to cytokine stimulus	0.002904263	GO:0006979	response to oxidative stress	0.00172093
GO:2000659	regulation of interleukin-1-mediated signaling pathway	0.002904263	GO:1903409	reactive oxygen species biosynthetic process	0.001843324
GO:2000660	negative regulation of interleukin-1-mediated signaling pathway	0.002904263	GO:0007268	chemical synaptic transmission	0.006462425
GO:1901575	organic substance catabolic process	0.003751786	GO:0098916	anterograde trans-synaptic signaling	0.006462425
GO:0002793	positive regulation of peptide secretion	0.005800392	GO:0099536	synaptic signaling	0.006462425
GO:0032024	positive regulation of insulin secretion	0.005800392	GO:0099537	trans-synaptic signaling	0.006462425
GO:0045766	positive regulation of angiogenesis	0.005800392	GO:0072593	reactive oxygen species metabolic process	0.007567269
GO:0000813	ESCRT I complex	0.014684033	GO:0005789	endoplasmic reticulum membrane	0.000111659

Table S6. KEGG enrichment the selected genes in BLM mink and SM mink.

BLM			SM		
**PathwayID**	**Pathway**	**Pvalue**	**PathwayID**	Pathway	Pvalue
ko00030	pentose phosphate pathway	0.094600521	ko04912	GnRH signaling pathway	0.128927223
ko00591	Linoleic acid metabolism	0.0881447	ko04211	Longevity regulating pathway	0.121923851
ko04950	Maturity onset diabetes of the young	0.084899894	ko04012	ErbB signaling pathway	0.108222608
ko04910	Insulin signaling pathway	0.077354749	ko03320	PPAR signaling pathway	0.094978685
ko04371	Apelin signaling pathway	0.072287196	ko04530	Tight junction	0.092417211
ko00790	Folate biosynthesis	0.071807136	ko04920	Adipocytokine signaling pathway	0.086434768
ko04270	Vascular smooth muscle contraction	0.068315842	ko04115	p53 signaling pathway	0.082255834
ko04611	Platelet activation	0.064421047	ko05206	MicroRNAs in cancer	0.06792953
ko00730	Thiamine metabolism	0.048451688	ko04014	Ras signaling pathway	0.066144013
ko04070	Phosphatidylinositol signaling system	0.043660546	ko02010	ABC transporters	0.038014768
ko04922	Glucagon signaling pathway	0.042826513	ko00270	Cysteine and methionine metabolism	0.036450049
ko00562	Inositol phosphate metabolism	0.026125144	ko00600	Sphingolipid metabolism	0.034911119
ko04360	Axon guidance	0.020218231	ko04624	Toll and Imd signaling pathway	0.029022037
ko00524	Neomycin, kanamycin and gentamicin biosynthesis	0.01757549	ko04310	Wnt signaling pathway	0.015687076
ko04930	Type II diabetes mellitus	0.011991785	ko04918	Thyroid hormone synthesis	0.010655342
ko04926	Relaxin signaling pathway	0.009375588	ko04062	Chemokine signaling pathway	0.006369985
ko04340	Hedgehog signaling pathway	0.008369162	ko04931	Insulin resistance	0.005710419
	alpha−Linolenic acid metabolism			Fc gamma R−mediated phagocytosis	
	AGE−RAGE signaling pathway in diabetic complications			MAPK signaling pathway − fly	
	Hedgehog signaling pathway − fly			Glycosaminoglycan biosynthesis − chondroitin sulfate / dermatan sulfate	
